# Seasonal Variations in Ocular Axial Length Increase among Children in the Czech Republic

**DOI:** 10.1155/2023/2592853

**Published:** 2023-02-10

**Authors:** Lenka Hecova, Stepan Rusnak, Vaclav Salcman, Jiri Cendelin

**Affiliations:** ^1^Department of Ophthalmology, Faculty of Medicine in Pilsen, Charles University in Prague and University Hospital in Pilsen, Prague, Czech Republic; ^2^Center of Eye Microsurgery Ofta, Pilsen, Czech Republic; ^3^Department of Physical Education and Sport, Faculty of Education, University of West Bohemia, Pilsen, Czech Republic; ^4^Department of Ophthalmology, 2nd Faculty of Medicine, Charles University in Prague and Motol University Hospital, Prague, Czech Republic

## Abstract

In recent decades, the prevalence of myopia has increased worldwide as well as in European countries, and it has become an important medical and socioeconomic problem. Our prospective single-center study analysed the changes in ocular axial length (AXL) in a population of Central European schoolchildren from 2016 to 2019. The study included 528 eyes of 264 children with a mean age of 12.2 years at the beginning of the study. Visual acuity, ocular AXL, anterior chamber depth, and the questionnaire were examined at 6-month intervals (in spring and autumn, following the winter and summer periods, respectively). The average ocular AXL was 23.329 mm (median: 23.315 mm) at the beginning of the study and 23.525 mm (median: 23.505 mm) at the end of the study. The change in ocular AXL per month was significantly higher (*p* < 0.0001) during the winter period (average: 0.013 mm, median: 0.011 mm) than during the summer period (average: −0.001 mm, median: 0.000 mm). We observed a significantly higher increase in ocular AXL in a Caucasian population during the winter period (with lower daylight exposure) than the summer period.

## 1. Introduction

Myopia is a refractive error caused by a disparity between the refractive index and AXL. The prevalence of myopia has increased worldwide in recent decades, and it has become an important medical and socioeconomic problem [1, 2]. Pathological myopia occurs when an increase in ocular AXL is associated with the development of pathologies of other ocular structures, especially the vitreous, retina, choroid, and optic nerve. These pathologies can endanger visual function, often uncontrollably and at a productive age. Prevention of myopia development is essential to stop the chain of serious pathological changes. The onset and progression of myopia are influenced by genetics and ethnicity [3–5]. A family history of myopia and Asian ethnicity are known risk factors. However, these factors alone cannot fully explain the increase in the prevalence of myopia in recent decades. Recent studies [6–8] have revealed urbanization as a possible risk factor. Urbanization manifests mainly by significantly reducing the time spent by children on physical outdoor activities and by increasing near-work activities. The external factors influencing the progressive development of myopia have been discussed in studies from Southeast Asia and Australia [9–13]. An association between the educational system and myopia has also been described [10, 14, 15]. The role of daylight exposure and outdoor activities in the prevention of the development and progression of myopia has been discussed in many papers, and it has been reported that an increase in the time spent outdoors was associated with a decrease in the prevalence of myopia [1, 9, 16]. However, the exact duration of outdoor activities required for this effect remains a key question in recent studies [9, 16]. Urbanization, educational systems, and the availability of entertainment through electronic devices, together with reduced outdoor physical activity and other influences, have collectively led to the development of a myopia epidemic in the developed regions of Southeast Asia [6, 8–10, 17]. These factors have led to a myopia prevalence of 96% among South Korean conscripts over several generations, leading to national security implications [14].

As mentioned earlier, most of the topics related to the risk factors for the development of myopia have been discussed in studies from Southeast Asia. Therefore, studies from other geographical and social areas are necessary. The prevalence of myopia in children and adolescents has increased in recent decades in European countries as well. In 2016, our team started a prospective study to analyse the changes in ocular AXL in a Central European population of schoolchildren. To evaluate the influence of daylight exposure, regular half-year examinations were performed in spring and autumn, following the winter (lower daylight exposure) and summer (higher daylight exposure) periods, respectively.

## 2. Group of Patients

This prospective study commenced in the spring of 2016 (April). The study included 528 eyes of 264 junior students from grammar schools in the city of Pilsen, Czech Republic. The children included in the study constituted a homogeneous group living in the same geographical location, belonged to the Europoid (Caucasian) race, had similar dietary habits, and were exposed to a similar level of environmental pollutants.

The study included children at the age of 10.9–13.6 years at the start of the study. The mean age was 12.2 years (median 12.2 years). The group consisted of 42.42% boys and 57.58% girls (Table 1).

At the beginning of the study, corrected myopia was detected in 280 eyes, i.e. in 53% cases.

## 3. Methodology

The children enrolled in the study were examined at 6-month intervals in April (spring) and October (autumn). For this study, data from eight examinations were used (01: spring 2016, 02: autumn 2016, 03: spring 2017, 04: autumn 2017, 05: spring 2018, 06: autumn 2018, 07: spring 2019, and 08: autumn 2019). We focused on monitoring the seasonal changes in ocular AXL. Therefore, individual measurements were distributed evenly within the year (every 6 months, always in spring and autumn). These periods included the whole summer with mid-spring and mid-autumn (period between examinations 01 and 02: summer 01, between examinations 03 and 04: summer 02, between examinations 05 and 06: summer 03, and between examinations 07 and 08: summer 04) and the whole winter with mid-spring and mid-autumn (period between examinations 02 and 03: winter 01, between examinations 04 and 05: winter 02, and between examinations 06 and 07: winter 03).

During each examination, the uncorrected visual acuity of both the eyes was determined using the Early Treatment of Diabetic Retinopathy Study logMAR optotypes at a distance of 4 m. The lighting intensity of the room was 50–100 lm/ft^2^. Examination of visual acuity was performed in a sitting position without correction (without glasses or contact lenses), and the fellow eye was covered with an eye patch or pad. The examination was performed in the schools. In these terrain conditions, the best-corrected visual acuity could not be determined.

Subsequently, ocular biometry measurements of both the eyes were performed using relevant techniques. For our research, the ocular AXL (measured in mm; the final value was the average of three measurements) and anterior chamber depth (measured in mm; the final value was an average of five measurements) of both the eyes were determined. Measurements were performed in a noncontact manner using IOL Master XP equipment (Carl Zeiss Meditec, Jena, Germany).

Finally, a questionnaire survey was conducted. The introductory part of the questionnaire focused on the eye (especially the history of eye surgery and the history of wearing glasses) and family history (history of refractive errors and other eye diseases in the parents). The next part of the questionnaire focused on the analysis of leisure time, including the time spent on near work activities (reading and working on mobile electronic devices, especially smartphones and tablets), time spent outdoors in the daylight, and time spent on sports activities. The questionnaire was updated regularly.

The research was conducted in cooperation with experts from the fields of physical education and prevention, who continuously evaluated the data and prepared preventive programs for preschool children. The study was approved by the Ethics Committee of the University Hospital, Pilsen. Parents of all children included in the study signed an informed consent form.

This paper deals with the problematic of AXL changes in different seasons of the years (summer and winter period, respectively), therefore, in the results we report the data related to this area only.

## 4. Statistical Analyses

Statistical analyses were performed using SAS SW (SAS Institute Inc.; Cary, NC, USA) and SW Statistica (StatSoft, Inc., Tulsa, OK, USA).

Basic statistical data such as mean, standard deviation, variance, median, minimum, and maximum were calculated for the measured parameters in the whole set and in individual groups and subgroups. Frequencies were examined for categorical variables. Selected statistical data were also processed graphically into box-and-whisker plot diagrams, histograms, and pie charts.

A nonparametric analysis of variance (ANOVA) (Wilcoxon two-tailed test or Kruskal–Wallis test) was used to compare the differences in the distributions of individual parameters between different groups. The changes in ocular AXL over time were examined using the paired Wilcoxon signed-rank test and Friedman's ANOVA. A repeated ANOVA was used to evaluate the changes in ocular AXL in relation to the data from the questionnaire. Multivariate analysis was performed using logistic regression analysis. The statistical significance was set at *α* = 5% (*p* value 0.05).

## 5. Results

### 5.1. Changes in Ocular AXL over Time

**Figure 1:**
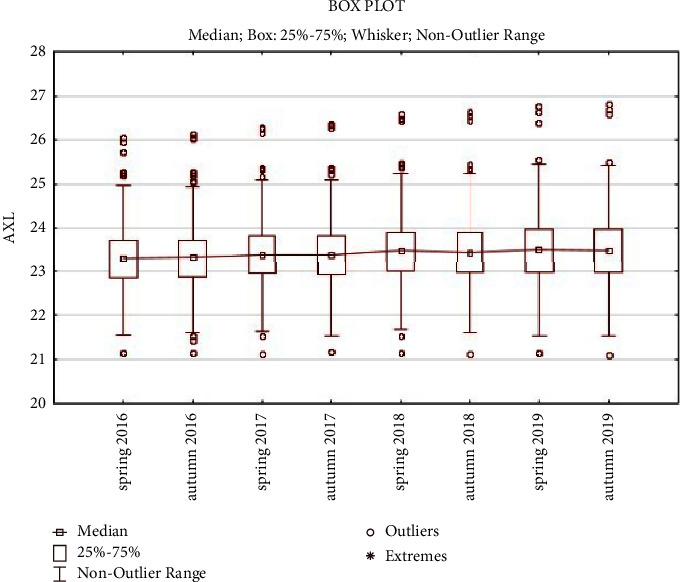
AXL-value development.

The average ocular AXL values were 23.329 mm at examination 01, 23.336 mm at examination 02, 23.406 mm at examination 03, 23.415 mm at examination 04, 23.496 mm at examination 05, 23.476 mm at examination 06, 23.541 mm at examination 07, and 23.525 mm at examination 08 (Table 2, Figure 1). The results confirmed that there was an increase in ocular AXL over time. An increase in ocular AXL between examinations 01 and 08 was observed in 89.55% of the eyes.

### 5.2. Seasonal Changes in Ocular AXL

The average change in ocular AXL during the summer of 2016 was 0.007 mm and the average change in ocular AXL per month was 0.001 mm, the change in ocular AXL during this period was not statistically significant (*p* < 0.1069). The average change in ocular AXL during the winter of 2017 was 0.071 mm and the average change in ocular AXL per month was 0.013 mm, the change in ocular AXL (increase) during this period was statistically significant (*p* < 0.0001). The average change in ocular AXL in the summer of 2017 was 0.009 mm and the average change in ocular AXL per month was 0.002 mm; the change in ocular AXL (increase) during this period was statistically significant (*p* < 0.0052). The average change in ocular AXL in the winter of 2018 was 0.081 mm and the average change in ocular AXL per month was 0.014 mm; the change in ocular AXL (increase) during this period was statistically significant (*p* < 0.0001). The average change in ocular AXL during the summer of 2018 was −0.019 mm and the average change in ocular AXL per month was −0.003 mm; the change in ocular AXL (decrease) during this period was statistically significant (*p*=0.0004). The average change in ocular AXL in the winter of 2019 was 0.064 mm and the average change in ocular AXL per month was 0.011 mm, the change in ocular AXL (increase) during this period was statistically significant (*p* < 0.0001). The average change in ocular AXL during the summer of 2019 was −0.015 mm and the average change in ocular AXL per month was −0.003 mm, the change in ocular AXL (decrease) during this period was statistically significant (*p* < 0.0004). (Tables 3 and 4).

**Figure 2:**
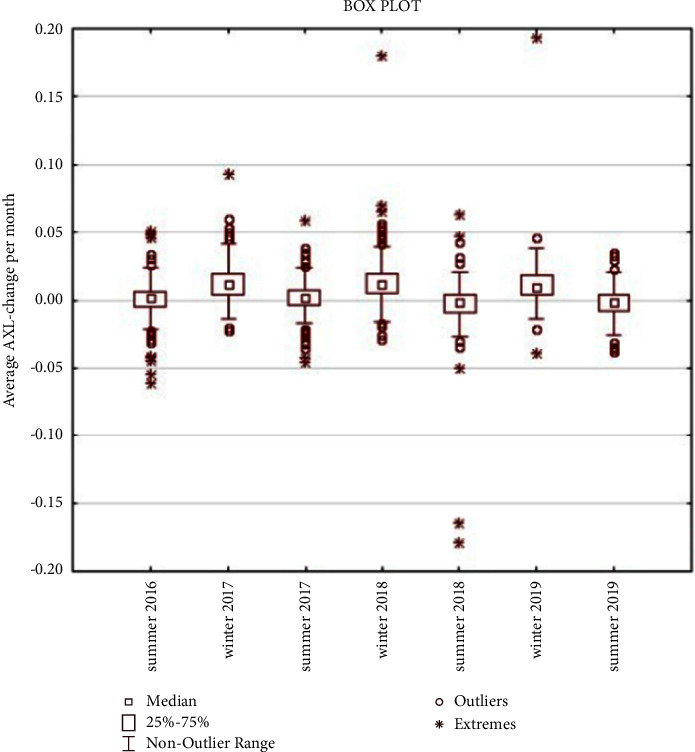
Average AXL change per month.

**Figure 3:**
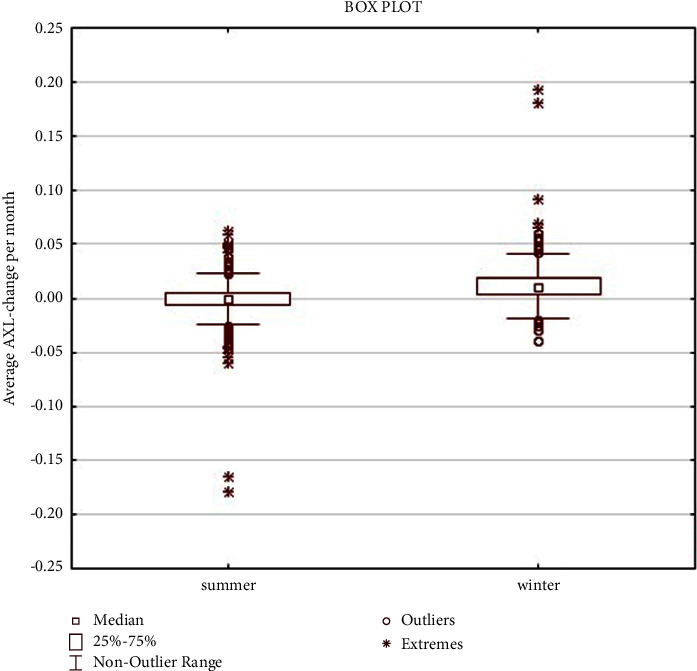
Average AXL-value per month in summer and winter seasons.

These results indicate that the increase in ocular AXL during the winter period (average change in ocular AXL: 0.072 mm and average change in ocular AXL per month: 0.013 mm) was significantly higher (*p* < 0.0001) than that in the summer period (average change in ocular AXL: −0.005 mm and average change in ocular AXL per month: −0.001 mm) (Tables 5 and 6; Figures 2 and 3).

**Figure 4:**
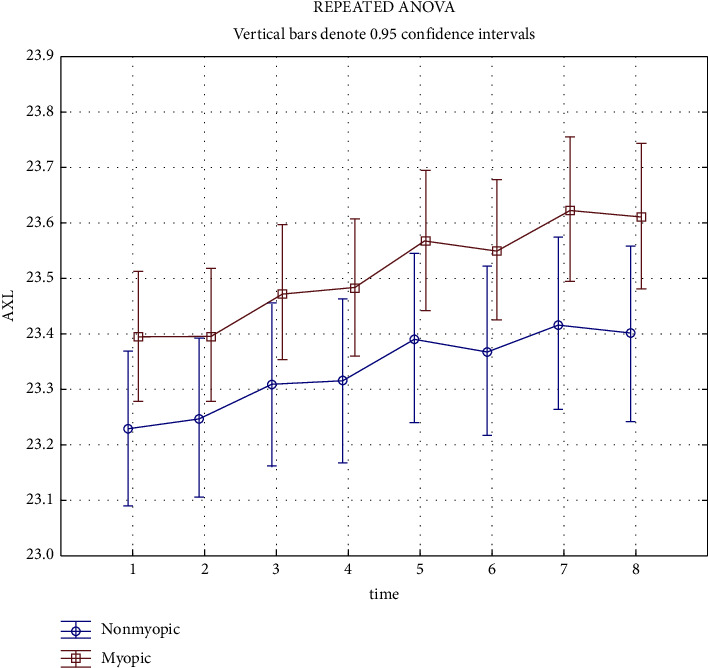
AXL-value developments in myopic and nonmyopic subgroups.

There was the average AXL of 23.397 mm at examination 01 and 23.612 mm at examination 08 in the subgroup of children with corrected myopia, and 23.230 mm at examination 01 and 23.401 mm at examination 08 in the subgroup of children without myopia. When comparing the AXL development in both groups over time (Figure 4), a statistically significant difference between the groups at time 01 was evident, which slightly but statistically insignificantly increased during the follow-up period.

## 6. Discussion

Myopia in children is an important worldwide medical and socioeconomic problem with a high risk of developing complications [18]. Influencing the incidence and progression of myopia is the current focus of many papers [1, 2, 6–9, 17, 19–21]. Insufficient daylight exposure is an independent risk factor for pathological AXL increase [1, 7, 9, 16, 22–24].

Various methods for stratifying the intensity of light have been described [25]. In these experiments, the intensity of light was easily and accurately measurable. However, in population and epidemiological studies, it is a difficult task and often poorly managed, which can lead to a fatal bias in the quality of results and their interpretation. Studies that examined the relationship between light intensity and the onset and progression of myopia used wearable electronic devices, known as exposure meters, to determine light intensity. These devices can accurately record light intensity over a particular period [25]. Apart from the high economic and logistical demands posed by these devices, these studies had limitations such as a short study length and a small number of participants (the maximum number of participants was 67 children in a study that used a wearable detector) [26].

More often, studies stratify the level of light exposure using questionnaires wherein the children are divided into predetermined categories by themselves, their parents, or examiners. These are often time-limited sections during which children typically perform certain activities, such as outdoor activities in nature, breaks between classes, or outdoor sports activities. In a pilot study [27], we verified that this form of input data stratification is inaccurate, and the possibility of subjective error by the respondents, which might be repeated throughout the study, is high. Neither the child nor the parents can accurately estimate the amount of time the child spends while performing a defined activity.

An interesting way of dividing the patients into groups based on the time spent under different light intensities was presented in a study comparing corresponding groups of Chinese children living in Sydney and Singapore [28]. The only input value that differentiated these two subgroups was the time spent outside: 13.5 hours in Sydney and 3.5 hours in Singapore. The children in Sydney exhibited a significantly lower prevalence of myopia. A Brazilian study [29] compared the initial ocular AXL of children living in the equatorial region (Aracati region) with that of children from the Campinas region located at 23° southern latitude (421 eyes, mean age at the beginning of the study: 10.6 years) and documented a shorter ocular AXL in children from Aracati, which has greater sun exposure.

Another way of distinguishing different exposures to daylight is to compare the values of refraction or changes in ocular AXL during the winter and summer periods in geographical regions lying between the Tropic of Cancer and the Arctic Circle (or between the Tropic of Capricorn in the Southern Hemisphere and the Antarctic Circle). Due to the tilt of the Earth's axis, the total light exposure in these regions naturally differs depending on the position of the Earth in the orbit around the sun [30]. Another factor that increases the possibility of daylight exposure is the organization of the school year, which has a long period of summer holidays [31]. The Czech Republic lies between 48° and 51° north latitudes. At 50° north latitude, daylight lasts for 13 h and 30 min to 16 h and 15 min during the period of July-August. During the Christmas holidays in December, daylight lasts only for 8 h and 5 min to 8 h and 15 min [30]. Therefore, there is a considerable difference between the exposure to natural light during summer (which also includes 2 months of summer holidays) and that during winter. Due to the distribution of teaching time, schoolchildren are practically not exposed to natural daylight during some weeks in the winter.

In the recent literature, two relevant studies have discussed the seasonal variability in the progression of myopia and the increase in ocular AXL. The COMET study [32] performed a subanalysis of the effect of seasonal variability on the progression of spherical equivalent by comparing the spring and autumn measurements with a half-year adjustment (182.5 d). This 3.5-year study included 358 children of various ethnicities, such as African American, Asian, Caucasian, and Hispanic. Significant progression of myopia was observed during the winter among children from all ethnic groups except Asians. Another similar study [33] examined seasonal changes in refraction and axial bulb length in 85 Chinese children. The study duration was only 1 year and the results showed higher progression of myopia and axial bulb length during the winter and among Asian children.

Our study included 264 children at the beginning of the study and statistical analysis was performed on 396 eyes of 198 children. The study included Caucasian children with different refraction statuses at the beginning of the study (emmetropic, hypermetropic, and myopic). Education and intensity of near work activities are considered separate risk factors for the onset and progression of myopia [10, 14, 34]. To reduce the input error as much as possible, a homogeneous group of patients was selected, which included children studying in the same year at a same school type (high school with an 8-year study program) in a single city. A similar homogeneous group has not yet been reported in the literature for studying the seasonal variability in the progression of myopia. An increase in ocular AXL over time was observed throughout the study. The mean initial ocular AXL of 23.4 mm was highly similar to that from a study with similar design, which included 12-year-old children from Brazil (23.29 mm). However, the mean initial ocular AXL was significantly lower than that the values reported in studies from China (23.85 mm) [35] and Japan (24.65 mm) [36], which included children of the same age.

We have already demonstrated seasonal variability in the rate of ocular AXL increase in our pilot study [27], wherein we performed three AXL measurements over a period of 1 year. While comparing the results from winter and summer, we found a statistically significant increase in ocular AXL during winter. A study comparing seasonal variability in the rate of ocular AXL increase over the same 1-year period (winter to summer) in Chinese children [33] showed a more pronounced ocular AXL increase during the winter.

To confirm long-term seasonal variability, we continued with the study and performed eight measurements. Thus, we obtained results for three winter periods and four summer periods. We documented a statistically significant increase in the average ocular AXL during winters and a statistically significant decrease in the average ocular AXL during two summer periods. No similar study in the literature has repeatedly demonstrated a significantly higher increase in ocular AXL during winters for 3.5 years.

Recent comprehensive reviews and meta-analyses [29] recommend the evaluation of cycloplegic refraction in epidemiological studies [37]. However, evaluation of cycloplegic refraction involves a partly invasive examination, which influences the visual acuity of children for several hours and is usually performed in medical facilities. In our study, data were collected directly from schools and in agreement with school management during classes. This method did not allow the examination in cycloplegia.

## 7. Conclusion

The worldwide prevalence of myopia has been proven by several population studies that do not hesitate to call it an epidemic. Currently, myopia is not just a medical problem, but is also becoming a serious socioeconomic threat. Two major risk factors were identified: level of education and reduced daylight exposure. Our research documented a significantly higher increase in ocular AXL in a Caucasian population during the winter periods (lower daylight exposure) than during the summer periods (higher daylight exposure). This result indicates that the aforementioned risk factors affect ocular AXL increases at a young age, before the typical occurrence of myopic changes in refraction. We believe that our study has contributed toward enhancing the understanding of the relationships among the risk factors for myopia, which is a serious diagnosis from both an epidemiological as well as individual point of view.

## Figures and Tables

**Table 1:** Group of patients.

Probands	Age at the study beginning (years)		Gender	
264	Mean	12.2	Female	152 (57, 58%)
	Median	12.2	Male	112 (42, 42%)

**Table 2:** AXL-values during the study.

Examination	Mean (mm)	Median (mm)
AXL-01	23.325	23.315
AXL-02	23.336	23.340
AXL-03	23.406	23.385
AXL-04	23.415	23.400
AXL-05	23.496	23.500
AXL-06	23.476	23.445
AXL-07	23.541	23.525
AXL-08	23.525	23.505

**Table 3:** AXL change in individual periods.

Period	Mean (mm)	Median (mm)
Summer 01	0.007	0.010
Winter 01	0.071	0.060
Summer 02	0.009	0.010
Winter 02	0.081	0.070
Summer 03	−0.019	−0.010
Winter 03	0.064	0.050
Summer 04	−0.015	−0.100

**Table 4:** AXL change per month in individual periods.

Period	Mean (mm)	Median (mm)
Summer 01	0.001	0.002
Winter 01	0.013	0.011
Summer 02	0.002	0.002
Winter 02	0.014	0.012
Summer 03	−0.003	−0.002
Winter 03	0.011	0.009
Summer 04	−0.003	−0.002

**Table 5:** AXL change in summer and in winter season.

Period	Mean (mm)	Median (mm)
Summer total	−0.005	0.000
Winter total	0.072	0.060

**Table 6:** AXL change per month in summer and in winter season.

Period	Mean (mm)	Median (mm)
Summer total	−0.0008	0.0000
Winter total	0.0125	0.0107

## Data Availability

The data used to support the findings of this study are available from the corresponding author upon request.
